# The comprehensive landscape of prognosis, immunity, and function of the GLI family by pan-cancer and single-cell analysis

**DOI:** 10.18632/aging.205630

**Published:** 2024-03-18

**Authors:** Yinteng Wu, Wenliang Guo, Tao Wang, Ying Liu, Marìa del Mar Requena Mullor, Raquel Alarcòn Rodrìguez, Shijian Zhao, Ruqiong Wei

**Affiliations:** 1 Department of Orthopedic and Trauma Surgery, The First Affiliated Hospital of Guangxi Medical University, Nanning, Guangxi 530021, China; 2 Department of Rehabilitation Medicine, Guigang City People’s Hospital, Guigang, Guangxi 537100, China; 3 Department of Orthopedics, The First Affiliated Hospital of Guangxi Medical University, Nanning, Guangxi 530021, China; 4 Department of Rehabilitation Medicine, The First Affiliated Hospital of Guangxi Medical University, Nanning, Guangxi 530021, China; 5 Faculty of Health Sciences, University of Almerìa, Almeria 04120, Spain; 6 Department of Cardiology, The Affiliated Cardiovascular Hospital of Kunming Medical University, Kunming, Yunnan 650102, China

**Keywords:** GLI, pan-cancer, single-cell, immune, prognosis, function

## Abstract

The Hedgehog (Hh) signaling pathway has been implicated in the pathogenesis of various cancers. However, the roles of the downstream GLI family (GLI1, GLI2, and GLI3) in tumorigenesis remain elusive. This study aimed to unravel the genetic alterations of GLI1/2/3 in cancer and their association with the immune microenvironment and related signaling pathways. Firstly, we evaluated the expression profiles of GLI1/2/3 in different cancer types, analyzed their prognostic and predictive values, and assessed their correlation with tumor-infiltrating immune cells. Secondly, we explored the relationships between GLI1/2/3 and genetic mutations, epigenetic modifications, and clinically relevant drugs. Finally, we performed enrichment analysis to decipher the underlying mechanisms of GLI1/2/3 in cancer initiation and progression. Our results revealed that the expression levels of GLI1/2/3 were positively correlated in most cancer tissues, suggesting a cooperative role of these factors in tumorigenesis. We also identified tissue-specific expression patterns of GLI1/2/3, which may reflect the distinct functions of these factors in different cell types. Furthermore, GLI1/2/3 expression displayed significant associations with poor prognosis in several cancers, indicating their potential as prognostic biomarkers and therapeutic targets. Importantly, we found that GLI1/2/3 modulated the immune microenvironment by regulating the recruitment, activation, and polarization of cancer-associated fibroblasts, endothelial cells, and macrophages. Additionally, functional enrichment analyses indicated that GLI1/2/3 are involved in the regulation of epithelial-mesenchymal transition (EMT). Together, our findings shed new light on the roles of GLI1/2/3 in tumorigenesis and provide a potential basis for the development of novel therapeutic strategies targeting GLI-mediated signaling pathways in cancer.

## INTRODUCTION

The adaptive and innate immune systems collaborate to create a sophisticated immune surveillance network that detects and eliminates abnormal cells carrying genetic mutations, thereby preventing the emergence of cancer. However, tumorigenesis driven by clonal selection and epigenetic changes involves multiple molecular mechanisms that ultimately culminate in the suppression of anti-tumor immunity and the evasion of tumor cells from immune recognition and elimination [1]. Remarkably, immune checkpoint inhibitors have demonstrated remarkable therapeutic efficacy by restoring the anti-tumor immune response in various metastatic cancers [2–4]. The emergence of cancer immunotherapy has revolutionized the field of oncology by providing a promising avenue for cancer treatment. Despite the remarkable effectiveness demonstrated in certain patients, the majority of patients exhibit intrinsic or acquired resistance to these interventions [5]. Numerous investigators are actively pursuing novel therapeutic approaches for the treatment of tumors [6, 7]. It is imperative, therefore, to elucidate the underlying mechanisms involved in cancer pathogenesis.

The Hedgehog (Hh) signaling pathway plays a crucial role in mammalian embryonic development, as well as in the growth and differentiation of cells following embryogenesis. Moreover, dysregulated activation of this pathway is implicated in the pathogenesis of malignant neoplasms affecting almost all human organ systems [8, 9]. GLI1, GLI2, and GLI3 are key downstream effectors of the Hh signaling pathway, acting as nuclear transcription factors that bind to promoters to regulate target gene expression. Originally discovered as highly expressed genes in human gliomas, the GLI family members all contain five C2H2 Krüppel-like zinc finger domains [10–12]. GLI1 contains only an activating C-terminal domain, whereas GLI2 and GLI3 possess both activating C-terminal domains and inhibitory N-terminal domains [13]. Despite sharing over 1,000 amino acids and functioning as zinc finger transcription factors, GLI1 functions primarily as a transcriptional activator, while GLI2 and GLI3 exhibit both activating and repressive properties [14]. GLI proteins are localized in both the nucleus and cytoplasmic compartments, where they are incorporated into a multimolecular complex that associates with the cytoskeleton [15].

GLI is implicated in the regulation of mammalian tumorigenesis by inducing factors associated with tumor stem cells, promoting cancer cell proliferation through cell cycle-related proteins, inhibiting apoptosis via direct binding to anti-apoptotic promoters, and upregulating Smad-interacting protein-1 (Sip1), which drives epithelial-mesenchymal transition (EMT). The expression of Sip1, an EMT-associated transcription factor regulator, plays a crucial role in tumor invasion and metastasis [14–18]. While studies investigating the role of GLI family genes in cancer have primarily focused on GLI1, the contribution of GLI2/3 to tumorigenesis, especially in the context of the tumor immune microenvironment (TIME), remains largely unknown. The TIME consists of a diverse array of immune components, including tumor cells, immune cells, and cytokines. The interplay among these immune constituents ultimately determines the immune status that can either promote or suppress tumor growth [19]. Extensive research has demonstrated the close association between TIME and various aspects of tumor development, recurrence, and metastasis. Activation of the Hh signaling pathway plays a pivotal role in modulating TIME, thereby influencing tumor initiation, progression, and metastasis [20]. Given the important role of GLI1/2/3 in the Hh signaling pathway. Systematically understanding their mechanisms in cancer and the immune microenvironment is necessary.

In this study, we conducted comprehensive analyses of the expression patterns, prognostic value, clinical characteristics, and genetic alterations of GLI1/2/3. We systematically investigated the mechanisms underlying their modulation of the tumor immune microenvironment, including immune infiltration, gene correlations, microsatellite instability (MSI), and tumor mutational burden (TMB). Furthermore, Gene Ontology (GO), Kyoto Encyclopedia of Genes and Genomes (KEGG), and Gene Set Enrichment Analysis (GSEA) were utilized to explore the biological processes affected by GLI1/2/3 in tumors. Finally, we identified potential drug targets that could simultaneously modulate these factors. Our results provide novel insights into the molecular mechanisms underlying the involvement of GLI1/2/3 in tumor development and their impact on the immune microenvironment. This study may also facilitate future research efforts focused on GLI1/2/3 as promising targets for cancer immunotherapy.

## RESULTS

### Correlation analysis

**Figure 1:**
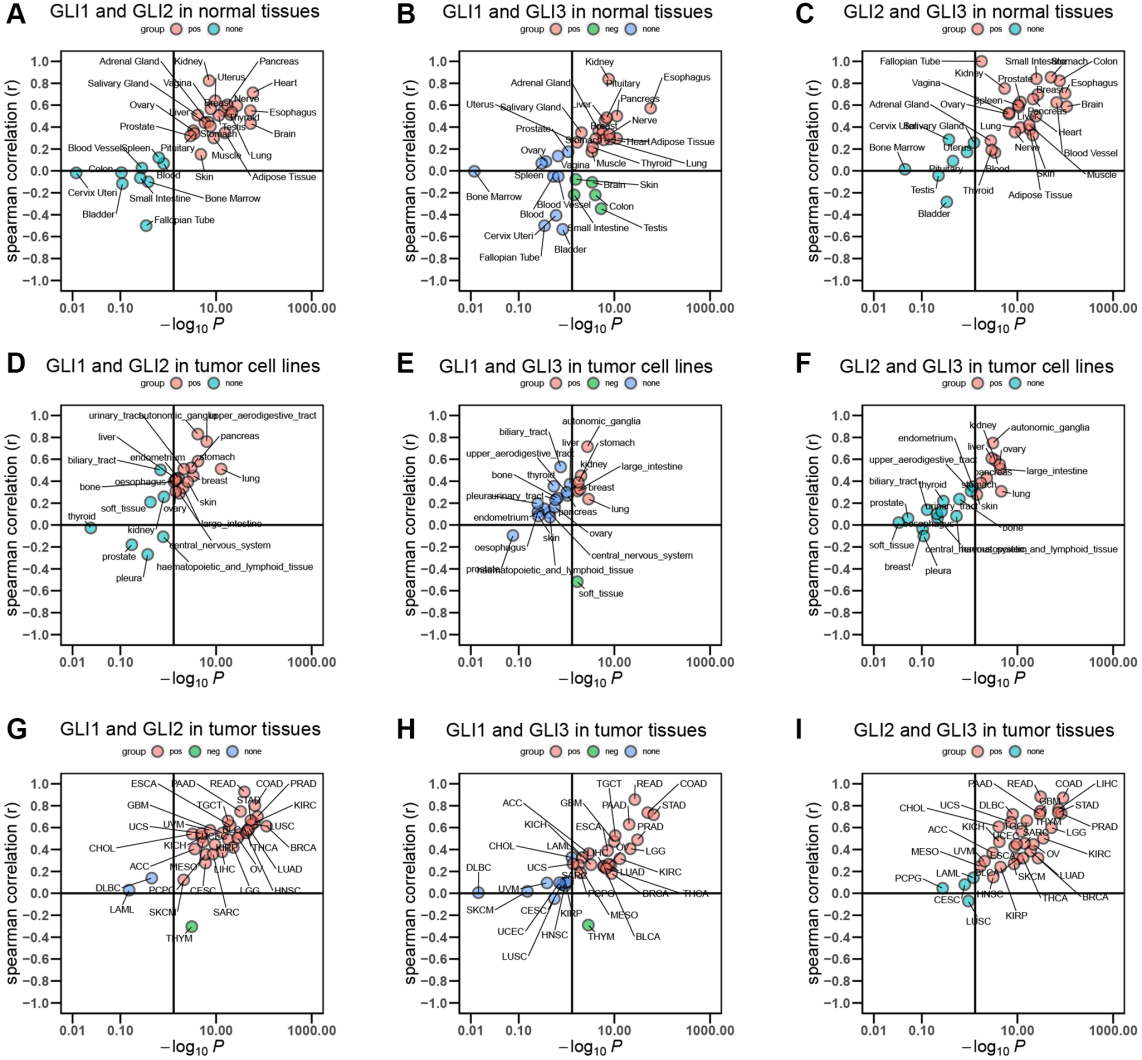
**Correlation between GLI1, GLI2, and GLI3.** Correlation of (**A**) GLI1 and GLI2, (**B**) GLI1 and GLI3, (**C**) GLI2 and GLI3 in normal tissues. Correlation of (**D**) GLI1 and GLI2, (**E**) GLI1 and GLI3, (**F**) GLI2 and GLI3 in tumor cell lines. Correlation of (**G**) GLI1 and GLI2, (**H**) GLI1 and GLI3, (**I**) GLI2 and GLI3 in tumor tissues.

Spearman correlation analyses of normal tissues from the GTEx database revealed that GLI1 and GLI2 exhibited a significant positive correlation in 22 of the examined tissues (Figure 1A). GLI1 and GLI3 also showed a significant positive correlation in 16 of the tissues, although in 5 cases the correlation was negative (Figure 1B). Meanwhile, GLI2 and GLI3 displayed a significant positive correlation in 24 of the analyzed normal tissues (Figure 1C). Similar analyses carried out on tumor cell lines indicated that GLI1 and GLI2 were significantly positively correlated in 15 of the tested cell lines (Figure 1D), while GLI1 and GLI3 exhibited significant positive correlations in 6 cell lines, but a negative correlation in 1 cell line (Figure 1E). Additionally, GLI2 and GLI3 displayed a significant positive correlation in 9 of the examined tumor cell lines (Figure 1F). Moreover, Spearman correlation analyses conducted on tumor tissues obtained from TCGA demonstrated significant positive correlations between GLI1 and GLI2 in 30 tumor tissues, with only one tissue showing a significant negative correlation (Figure 1G). GLI1 and GLI3 were significantly positively correlated in 21 tumor tissues but negatively correlated in one tissue (Figure 1H). Finally, GLI2 and GLI3 exhibited a significant positive correlation in 29 tumor tissues (Figure 1I).

### Expression level analysis results

**Figure 2:**
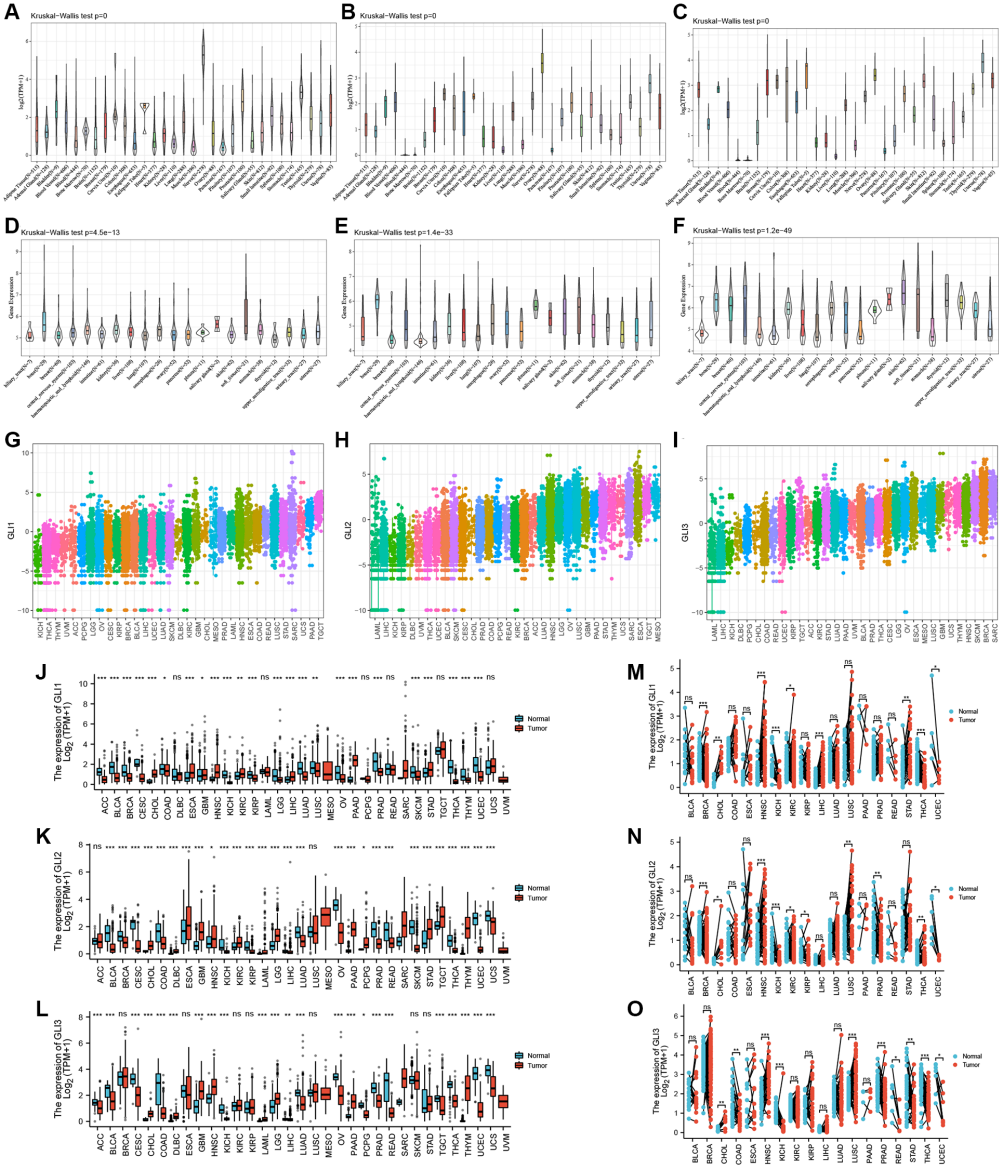
**Expression profiles of GLI1, GLI2, and GLI3.** Expression levels of GLI1, GLI2, and GLI3 in normal tissues (**A**–**C**), tumor cell lines (**D**–**F**), and tumor tissues (**G**–**I**) using data from the GTEx database. Combined TCGA and GTEx data of GLI1 (**J**), GLI2 (**K**), and GLI3 (**L**) expression differences between tumor and normal tissues (^*^p < 0.05, ^**^p < 0.01, and ^***^p < 0.001; Abbreviation: ns: no significance). Differential expression of GLI1 (**M**), GLI2 (**N**), and GLI3 (**O**) between paired tumors and normal tissues using data from the TGGA database (^*^p < 0.05, ^**^p < 0.01, and ^***^p < 0.001; Abbreviation: ns: no significance).

**Figure 3:**
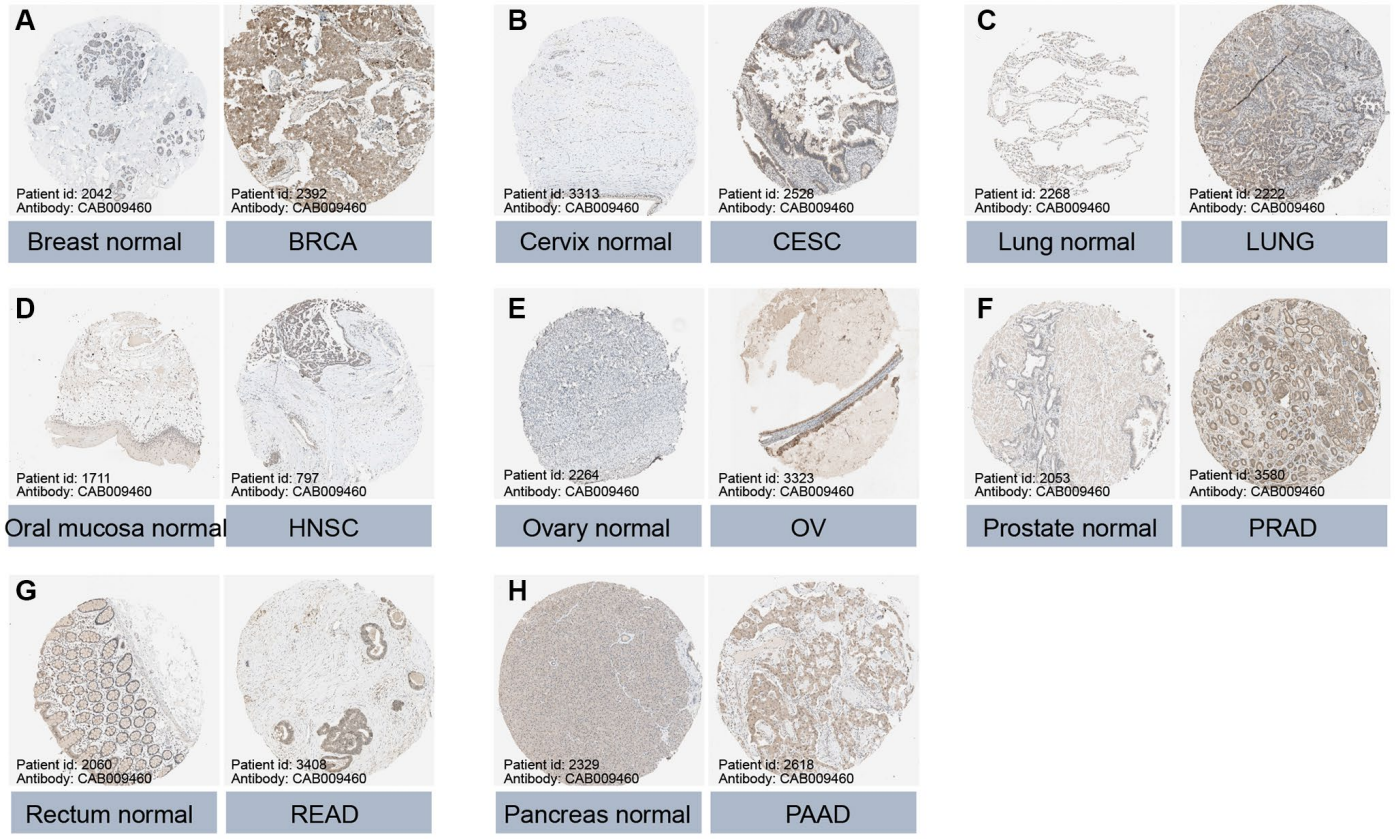
**Protein expression of GLI1 in normal and tumor tissues.** Immunohistochemical staining of normal and tumor tissues in the HPA database. (**A**) Breast normal, BRCA. (**B**) Cervix normal, CESC. (**C**) Lung normal, LUNG. (**D**) Oral mucosa normal, HNSC. (**E**) Ovary normal, OV. (**F**) Prostate normal, PRAD. (**G**) Rectum normal, READ. (**H**) Pancreas normal, PAAD.

**Figure 4:**
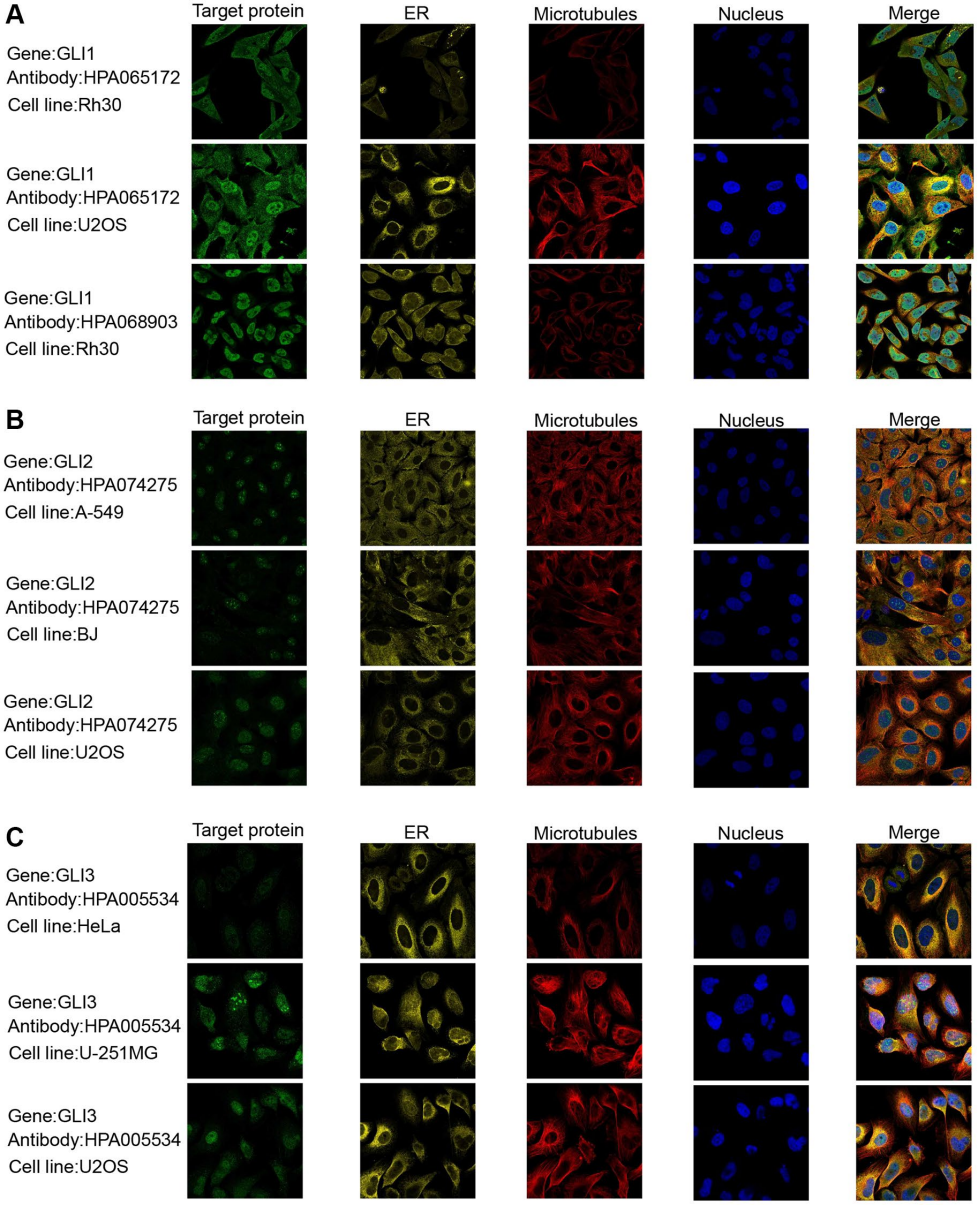
Protein subcellular localization the immunofluorescence images of (**A**) GLI1, (**B**) GLI2, and (**C**) GLI3 protein.

Data from the GTEx database revealed that GLI1 (Figure 2A) and GLI2 (Figure 2B) were most highly expressed in ovarian tissues, whereas GLI3 (Figure 2C) exhibited peak expression in uterine samples. In tumor cell lines, bone tumors displayed the highest expression of both GLI1 (Figure 2D) and GLI2 (Figure 2E), whereas skin tumor cell lines exhibited the greatest expression of GLI3 (Figure 2F). Regarding tumor tissues, TGCT, MESO, and SKCM exhibited the highest levels of GLI1 (Figure 2G), GLI2 (Figure 2H), and GLI3 (Figure 2I), respectively. Combining data from TCGA tumor samples and normal tissues from the GETx database showed significant expression of GLI1 (Figure 2J), GLI2 (Figure 2K), and GLI3 (Figure 2L) in 24, 28, and 23 types of cancer, respectively. Analysis of paired tumor and normal tissue samples from the TCGA database further verified significant expression of GLI1 (Figure 2M), GLI2 (Figure 2N), and GLI3 (Figure 2O) in nine, ten, and ten types of tumors, respectively. Immunohistochemistry (IHC) results for GLI1 in BRCA, CESC, LUNG, HNSC, OV, PRAD, READ, PAAD, and corresponding normal tissues are shown in Figure 3. In normal tissue, GLI1 IHC staining is negative or shows mild intensity. In tumor tissue, GLI1 IHC staining exhibits moderate to strong intensity. By performing immunofluorescence staining for GLI in Rh30 and U2OS cell lines, we obtained spatial localization and expression intensity information for GLI. Additionally, we conducted immunofluorescence staining for GLI2 in A-549, BJ, and U2OS cell lines, as well as for GLI3 in HeLa, U-251MG, and U2OS cell lines. Through these analyses, we were able to assess the spatial distribution and expression levels of these genes. The immunofluorescence staining provided insights into the localization and relative abundance of GLI, GLI2, and GLI3 proteins in the respective cell lines. Immunofluorescence (IF) images demonstrated that GLI1 was primarily located in the nucleoplasm, followed by the cytosol (Figure 4A). Meanwhile, GLI2 was predominantly found in the nucleoplasm and nucleoli (Figure 4B), and GLI3 was primarily found in the nucleoplasm, followed by nucleoli and vesicles (Figure 4C). Figure 3 (https://www.proteinatlas.org/search/GLI1) and Figure 4 (https://www.proteinatlas.org/search/GLI1, https://www.proteinatlas.org/search/GLI2, https://www.proteinatlas.org/search/GLI3) available from v23.0. https://www.proteinatlas.org.

### Survival analysis

**Figure 5:**
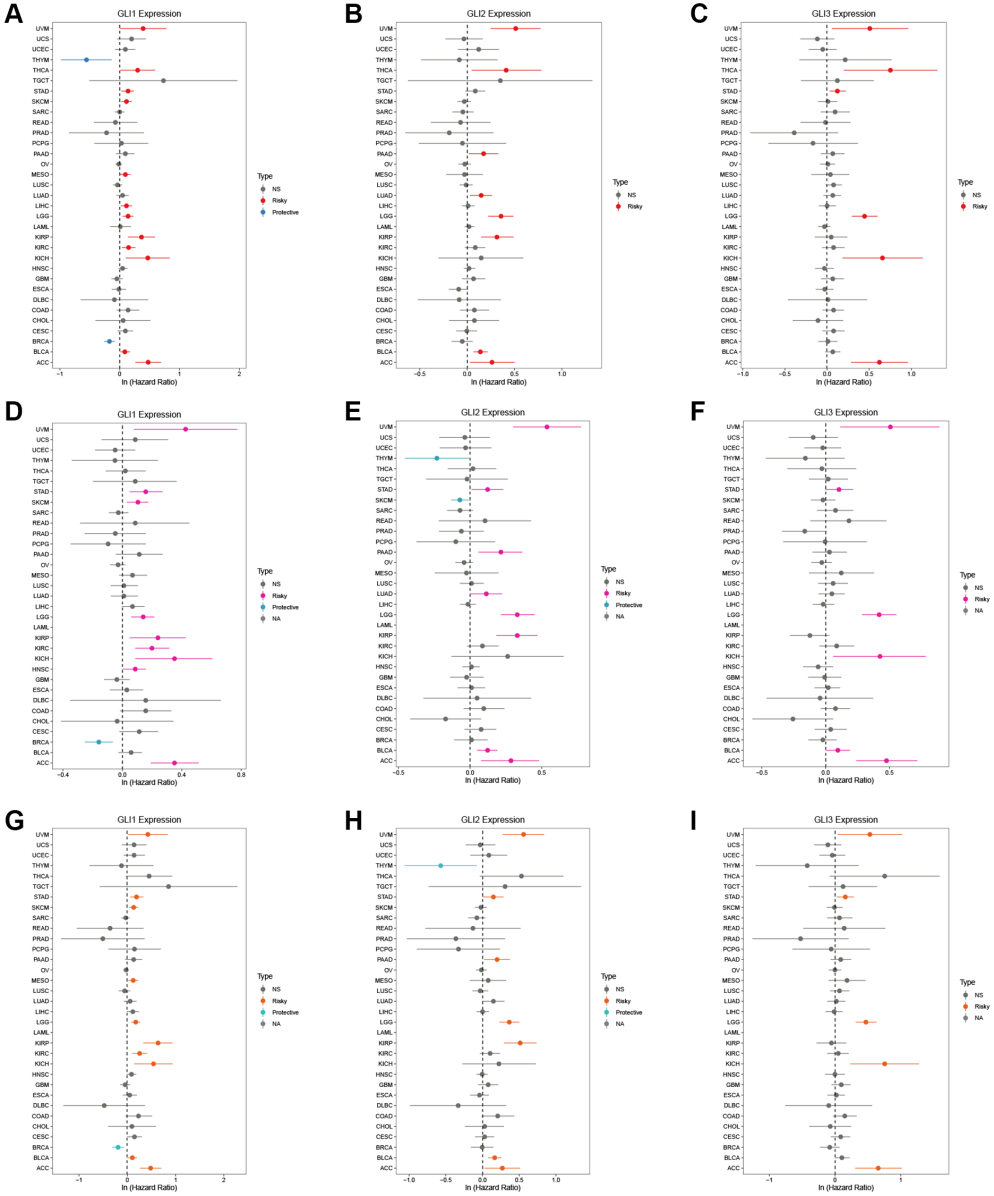
**Expression of GLI1, GLI2, and GLI3 with OS, PFI, and DSS in tumor patients.** Forest plots of hazard ratios of GLI1, GLI2, and GLI3 in OS (**A**–**C**), PFI (**D**–**F**), and DSS (**G**–**I**).

**Figure 6:**
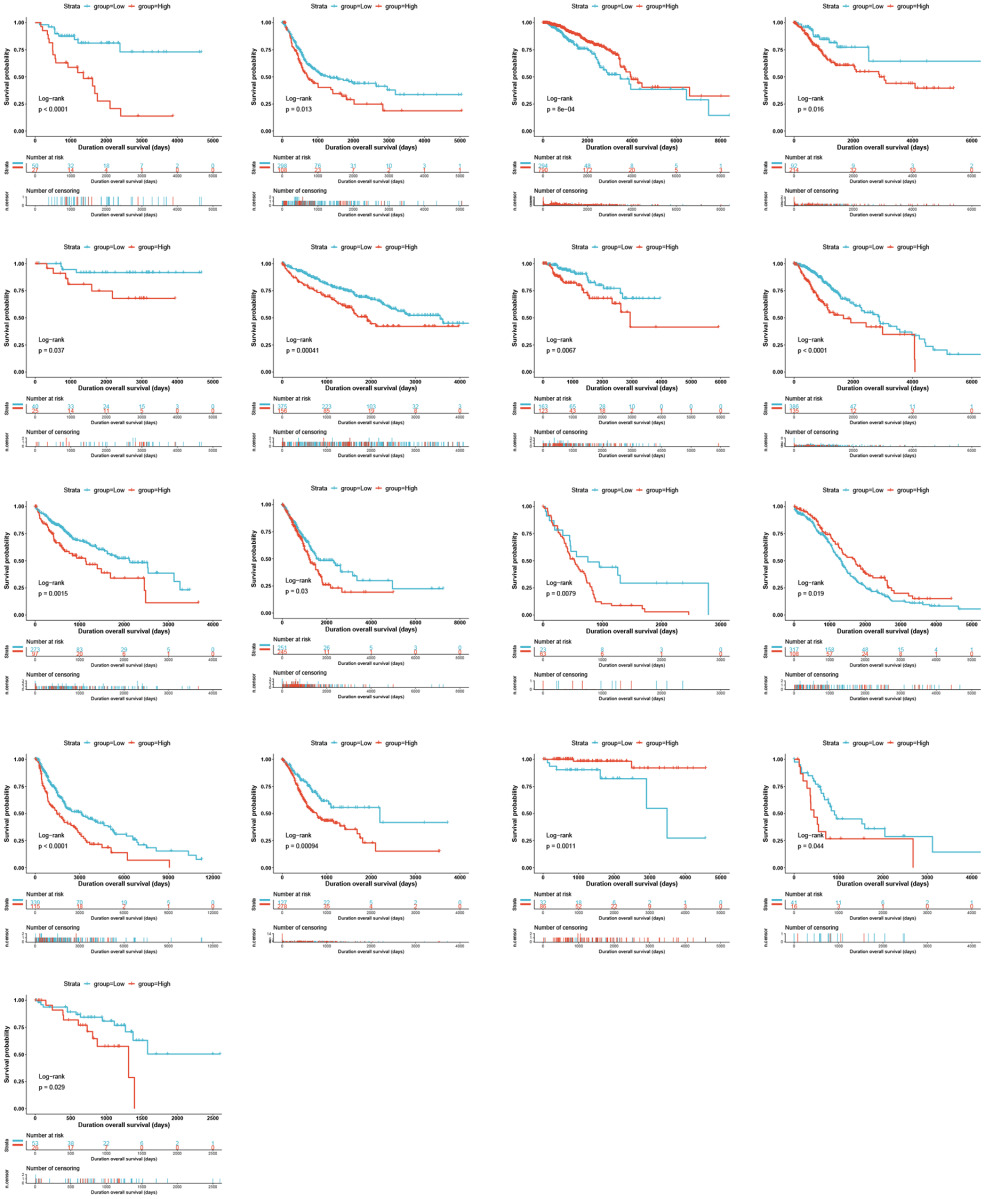
Kaplan–Meier OS curves for patients stratified by different expression levels of GLI1 in seventeen cancer types.

**Figure 7:**
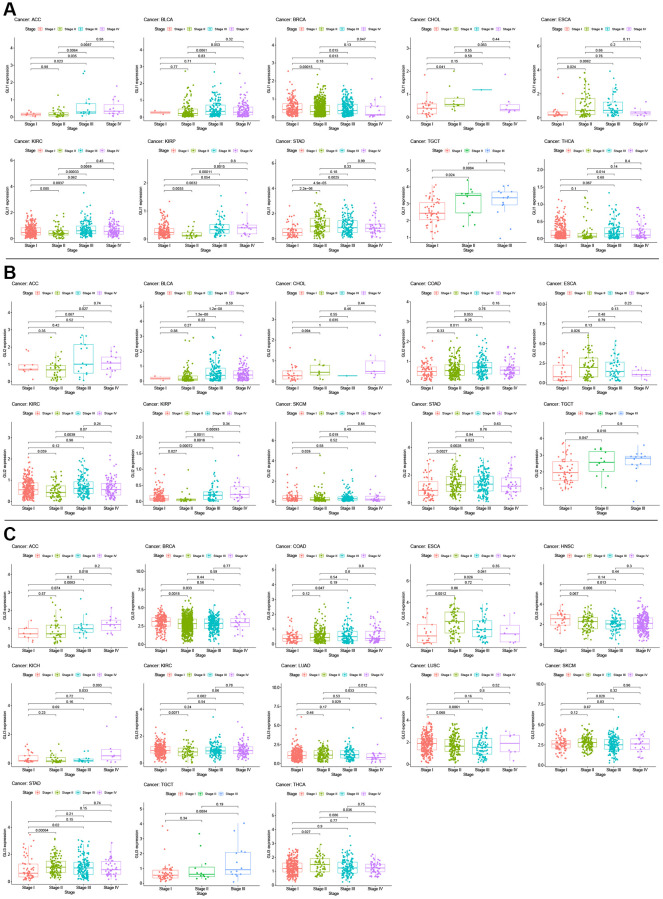
Correlation between GLI1 (**A**), GLI2 (**B**), and GLI3 (**C**) expression with clinical stage in cancer patients.

Univariate Cox analysis for overall survival (OS) demonstrated that GLI1 (Figure 5A), GLI2 (Figure 5B), and GLI3 (Figure 5C) were risk factors for patients with 12, 8, and 6 types of cancer, respectively. Similarly, GLI1 (Figure 5D), GLI2 (Figure 5E), and GLI3 (Figure 5F) were identified as prognostic risk factors for patients with 9, 8, and 6 types of cancer, respectively, using progression-free interval (PFI) analyses. Additionally, univariate Cox analysis of disease-specific survival (DSS) revealed that GLI1 (Figure 5G), GLI2 (Figure 5H), and GLI3 (Figure 5I) were risk factors for patients with 10, 7, and 5 types of cancer, respectively. Kaplan-Meier analysis of GLI1 (Figure 6) indicated that high expression of GLI1 was associated with decreased probability of survival in 14 types of tumors and increased probability in three types of tumors. Moreover, GLI1 (Figure 7A), GLI2 (Figure 7B), and GLI3 (Figure 7C) were significantly expressed in ten, ten, and thirteen tumor stages, respectively, suggesting their involvement in tumor progression.

### Genetic variation analysis

**Figure 8:**
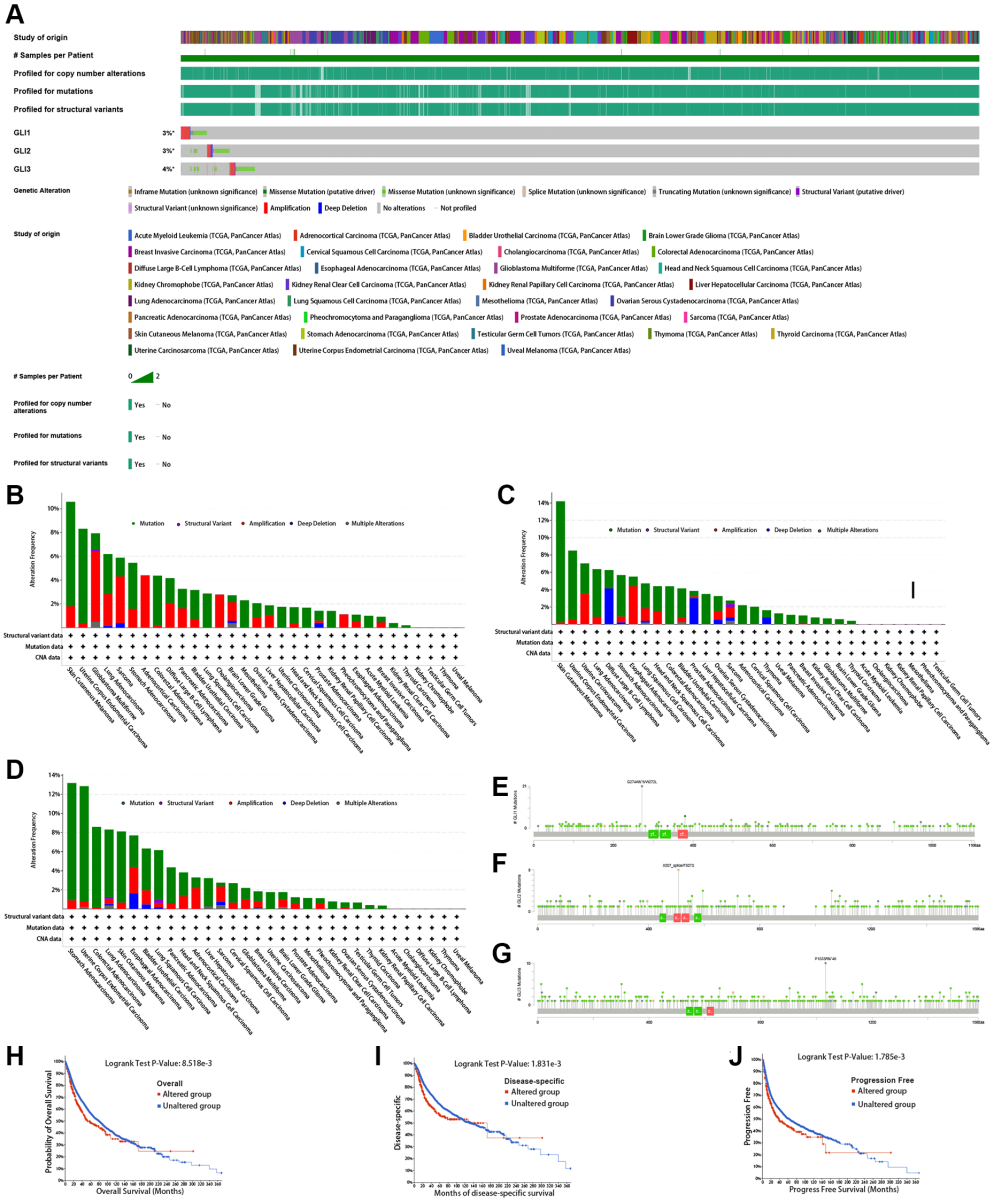
**Mutation signature mapping of GLI1, GLI2, and GLI3 genes in the cBioPortal database.** (**A**) An overview of the genomic alternations of GLI1, GLI2, and GLI3 occurred in pan-cancer. The mutation frequency and corresponding mutation types of GLI1 (**B**), GLI2 (**C**), and GLI3 (**D**) in different cancers. Mutation sites of GLI1 (**E**), GLI2 (**F**), and GLI3 (**G**). Kaplan-Meier plot showing the comparison of OS (**H**), DSS (**I**), and PFS (**J**) in cases with/without GLI1 gene alterations in the tumor.

**Figure 9:**
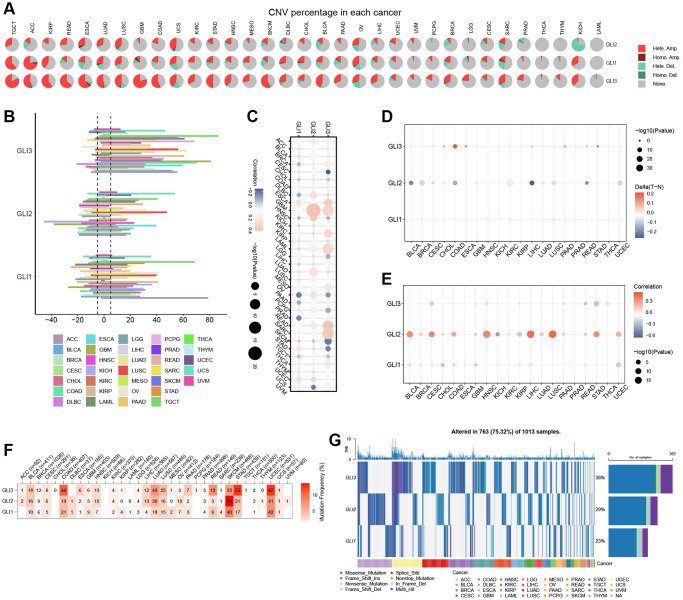
**Correlation analysis of GLI1, GLI2, and GLI3 with CNV, methylation, and mutation frequency.** (**A**) Type of genetic variation. (**B**) CNV expression of GLI1, GLI2, and GLI3 in human pan-cancer. (**C**) Correlation between CNV expression of GLI1, GLI2, and GLI3 and their mRNA expression. (**D**) Correlation between GLI1, GLI2, and GLI3 and methylation in various tumors. (**E**) Correlations between GLI1, GLI2, and GLI3 mRNA expression and methylation in various tumors. (**F**) The mutation frequency of GLI1, GLI2, and GLI3 in various tumors. (**G**) SNV oncoplot. An oncoplot showing the mutation distribution of GLI1, GLI2, and GLI3 and a classification of SNV types.

Figure 8A displays the mutation information for GLI1/2/3. The highest mutation rates were found in GLI1 (Figure 8B), GLI2 (Figure 8C), and GLI3 (Figure 8D) at approximately 10%, 14%, and 13%, respectively. Mutation site information for GLI1 (Figure 8E), GLI2 (Figure 8F), and GLI3 (Figure 8G) are also presented. Mutations in GLI1 were shown to affect patient survival, reducing the probability of OS (Figure 8H), DSS (Figure 8I), and progression-free survival (PFS) (Figure 8J). Hete amp was the most common copy number variation (CNV) type observed in tumors (Figure 9A). CNV expression levels of GLI1/2/3 are illustrated in Figure 9B, with correlation analysis revealing that mRNA expression levels of GLI1/2/3 were positively correlated with CNV expression levels (Figure 9C). Notably, while hypermethylation was primarily associated with GLI1 and GLI3, hypomethylation was primarily associated with GLI2 (Figure 9D). Figure 9E depicts the correlation between mRNA expression of GLI1/2/3 and methylation. Single-nucleotide variants (SNV) mutation rate analyses showed that GLI1 and GLI3 had the highest SNV mutation rates of 47% and 67%, respectively, in UCEC, whereas GLI2 exhibited a 64% SNV mutation rate in SKCM (Figure 9F). Finally, Figure 9G shows that GLI1/2/3 were mainly associated with missense mutations.

### Immuno-infiltration analysis

**Figure 10:**
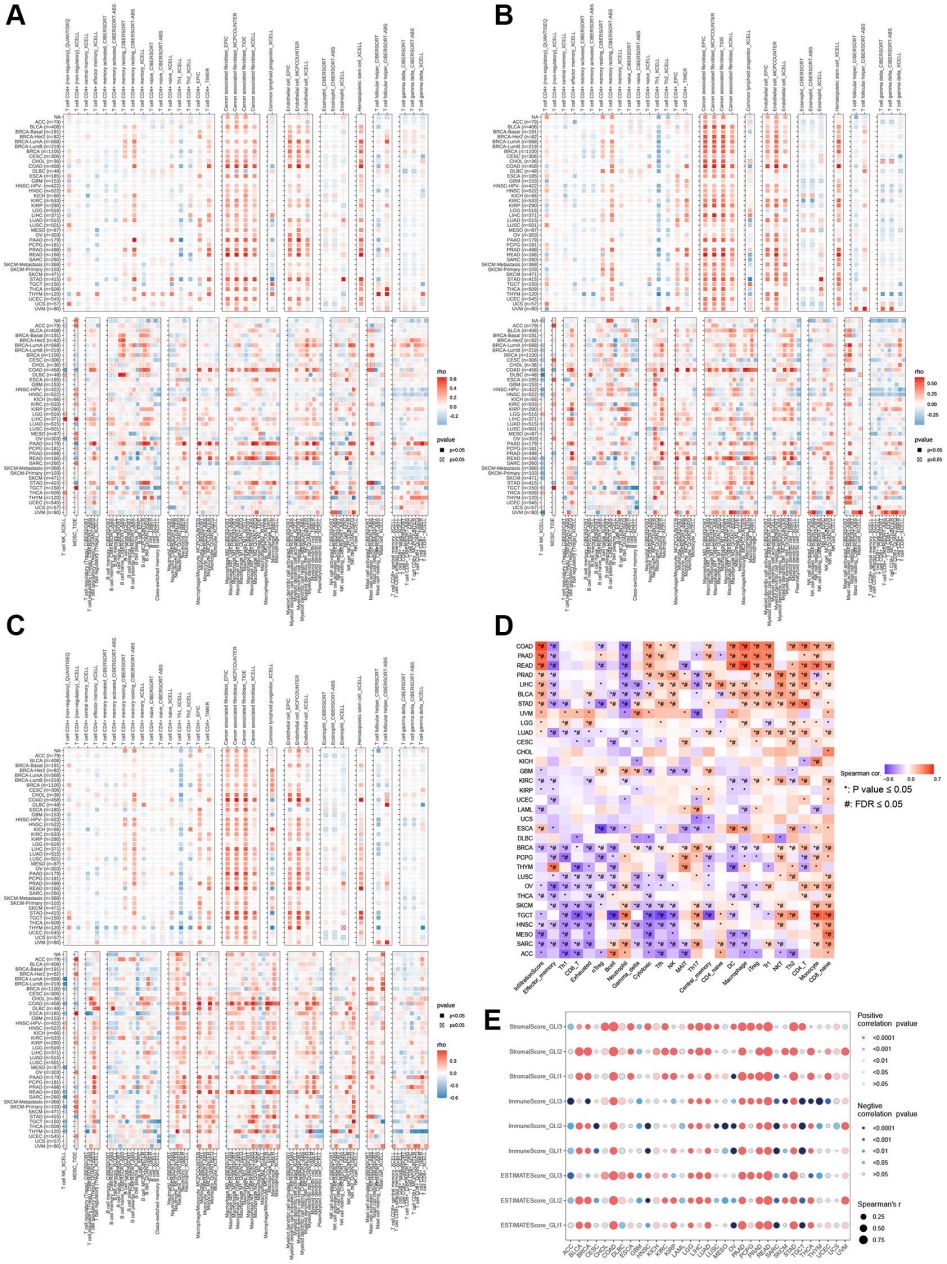
**Infiltration of immune cells in tumors.** Correlation of GLI1 (**A**), GLI2 (**B**), and GLI3 (**C**) with tumor-infiltrating immune cells according to different algorithms. (**D**) Correlation of GLI1, GLI2, and GLI3 with different species of immune cell subtypes. (**E**) Relationship between GLI1, GLI2, and GLI3 and three scores such as ESTIMATEScore, ImmuneScore, and StromalScore in different cancer types.

According to the correlation analysis of the TIMER2.0 database, in most tumors, fibroblasts, endothelial cells, and macrophages exhibited strong positive connections with GLI1 (Figure 10A), GLI2 (Figure 10B), and GLI3 (Figure 10C). Furthermore, gene set variation analysis (GSVA) scores for GLI1/2/3 demonstrated a positive correlation with CD8_naïve (Figure 10D). The correlation analysis results for GLI1/2/3 with StromalScore, ImmuneScore, and ESTIMATEScore are presented in Figure 10E, with significant positive correlations observed primarily.

### Correlation analysis

**Figure 11:**
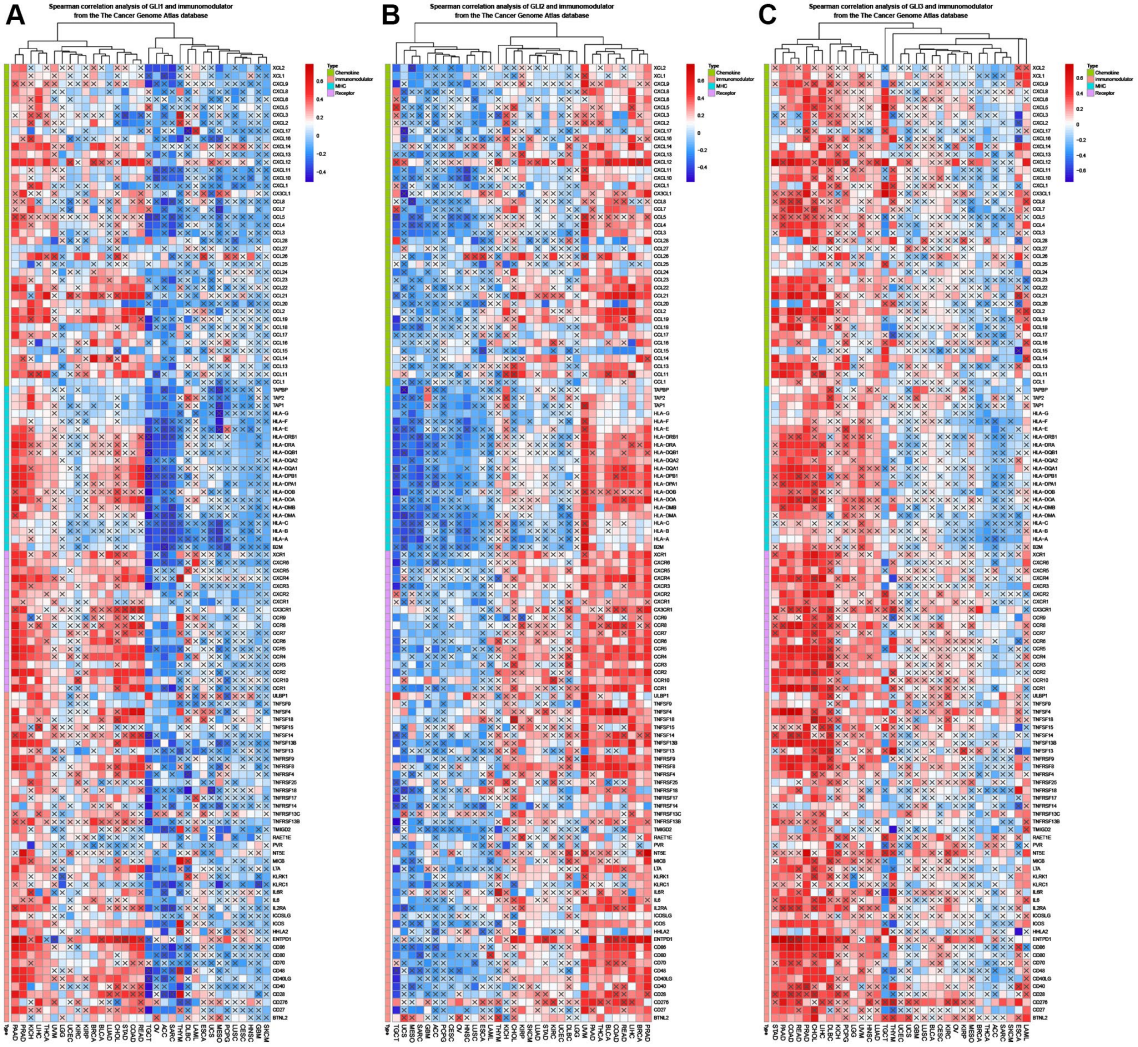
Coexpression of GLI1 (**A**), GLI2 (**B**), and GLI3 (**C**) with immune-related genes (X means *P* > 0.05).

**Figure 12:**
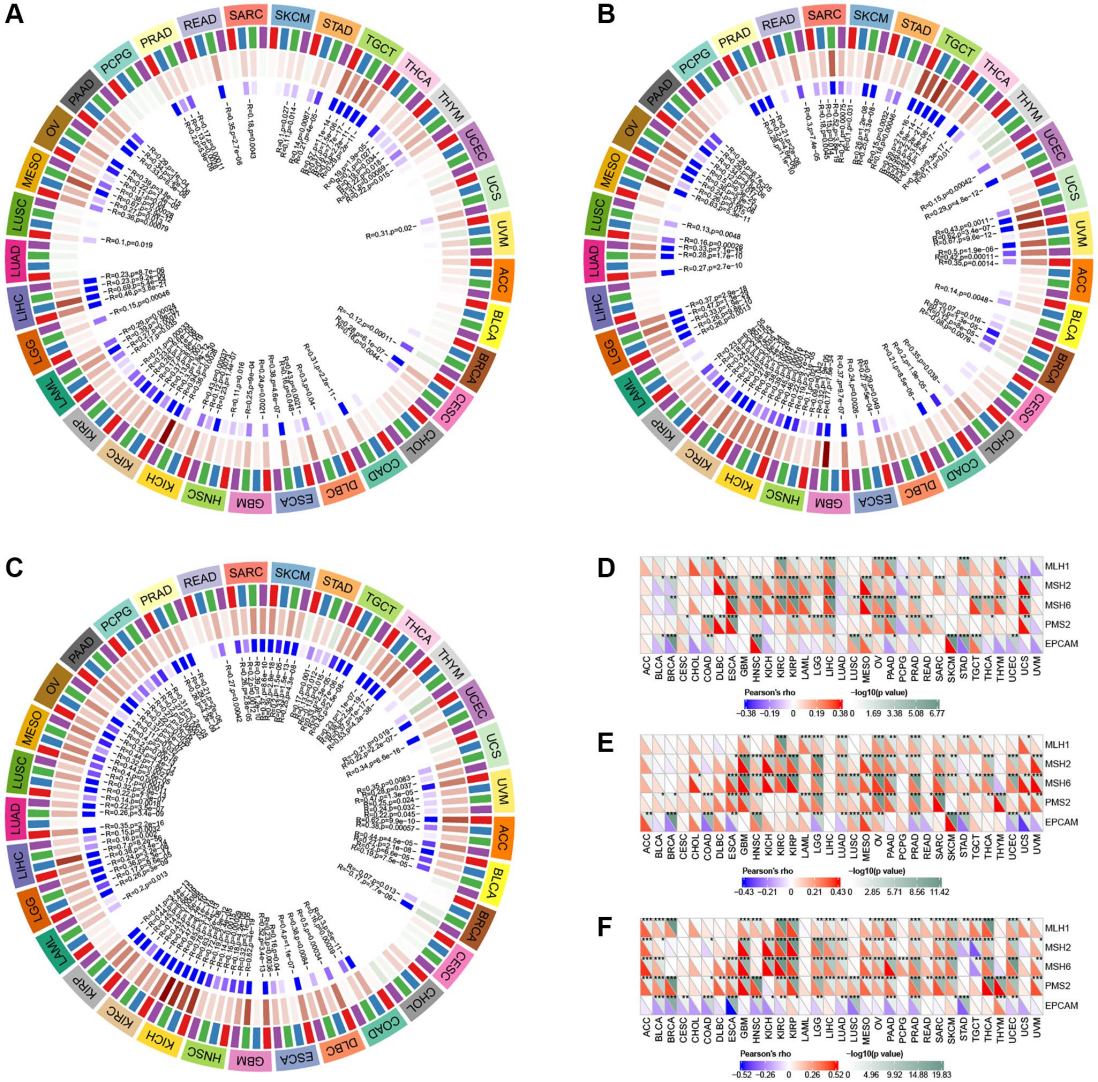
Correlation analysis of GLI1 (**A**), GLI2 (**B**), and GLI3 (**C**) expression with DNA methyltransferases (Red represents DNMAT1, blue represents DNMT2, green represents DNMT3A, and purple represents DNMT3B). Correlation analysis of GLI1 (**D**), GLI2 (**E**), and GLI3 (**F**) expression with MMRs genes in human pan-cancer (^*^P < 0.05, ^**^P < 0.01, ^***^P < 0.001).

In BLCA, BRCA, COAD, KIRP, LIHC, LUAD, LUSC, PAAD, PRAD, STAD, TGCA, and THAD, GLI1 was primarily associated with immune-related genes (Figure 11A), while in BLCA, BRCA, COAD, HNSC, KICH, KIRP, LIHC, LUAD, LUSC, PAAD, PRAD, STAD, TGCA, THAD, UVM, GLI2 was linked to immune-related genes (Figure 11B). In contrast, GLI3 was associated with immune-related genes in BRCA, LGG, LIHC, LUAD, LUSC, PAAD, PRAD, READ, and STAD (Figure 11C). GLI1 (Figure 12A), GLI2 (Figure 12B), and GLI3 (Figure 12C) were significantly positively correlated with methyltransferases, particularly GLI3. Correlation analysis of GLI1 (Figure 12D), GLI2 (Figure 12E), and GLI3 (Figure 12F) with mismatch repair (MMR) genes was also shown in the figure.

### TMB and MSI analysis

**Figure 13:**
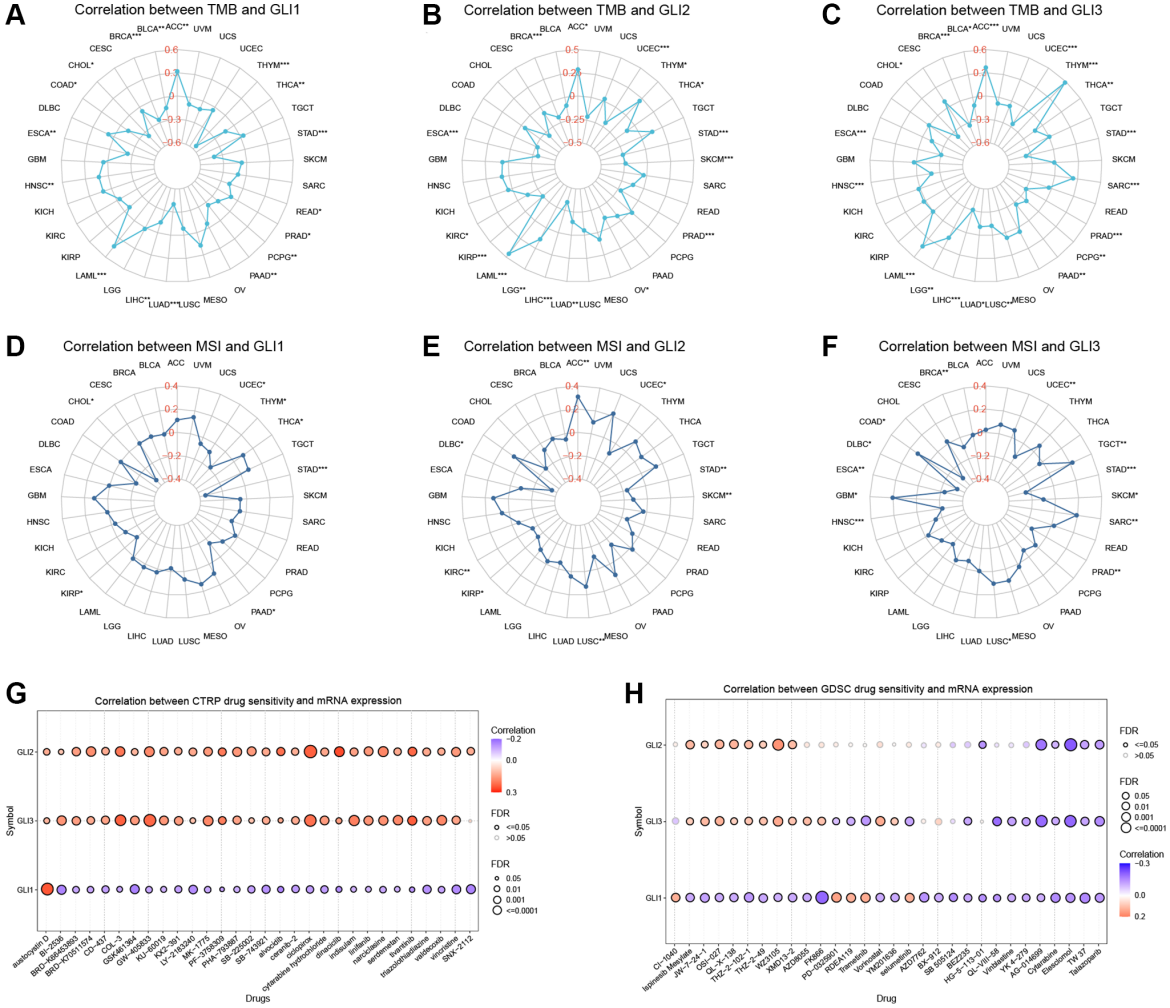
**Relationship of GLI1, GLI2, and GLI3 with TMB, MSI, and drug sensitivity.** The Relationship of GLI1, GLI2, and GLI3 expression with TMB (**A**–**C**) and MSI (**D**–**F**). Drug sensitivity analysis in CTRP database (**G**) and GDSC database (**H**).

Significant correlations were observed between GLI1 (Figure 13A), GLI2 (Figure 13B), and GLI3 (Figure 13C) and TMB in 17, 16, and 19 types of tumors, respectively. Similarly, significant correlations between GLI1 (Figure 13D), GLI2 (Figure 13E), and GLI3 (Figure 13F) and MSI were found in seven, eight, and thirteen types of tumors, respectively.

### Drug sensitivity analysis

Figure 13G presents the results from the CTRP database, demonstrating that GLI2 and GLI3 were significantly and positively correlated with 30 drugs, while GLI1 was significantly and positively correlated with only one drug. Similarly, the GDSC database revealed enhanced sensitivity or resistance to different drugs linked to GLI1/G2/3 (Figure 13H). Prognostic analysis of immunotherapy showed that in Kim cohort 2019 (Anti−PD−1/PD−L1) and IMvigor210 cohort 2018 (Anti−PD−L1), GLI1 (Supplementary Figure 1A, 1B), GLI2 (Supplementary Figure 1C, 1D), GLI3 (Supplementary Figure 1E, 1F), GLI1/23 gene set (Supplementary Figure 1G, 1H) were significant.

### Enrichment analysis

**Figure 14:**
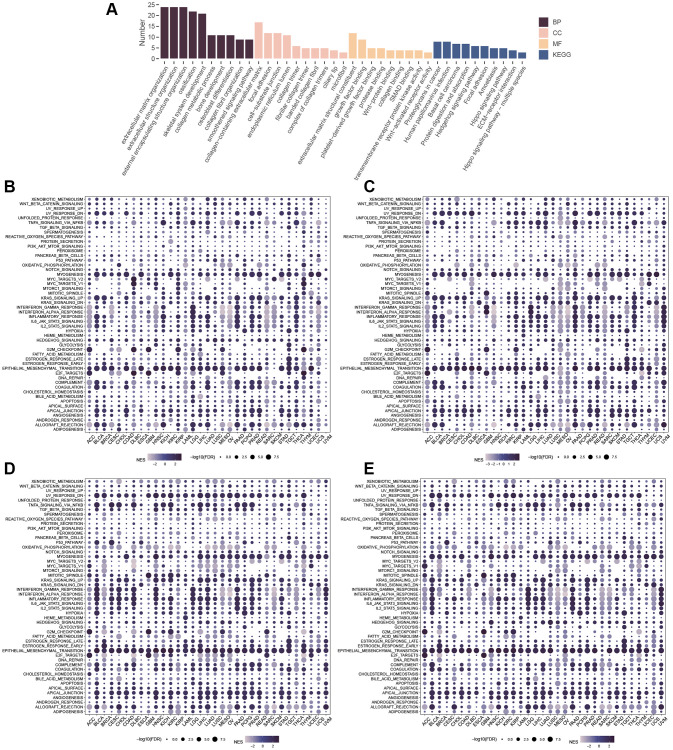
**Enrichment Analysis.** (**A**) GO and KEGG of similar genes. GSEA of GLI1 (**B**), GLI2 (**C**), GLI3 (**D**), and GLI1/2/3 gene sets (**E**).

Results from the GO analysis of similar genes revealed their association with extracellular matrix organization, extracellular structure organization, and external encapsulating structure organization (Figure 14A), with related terms including collagen-containing extracellular matrix, focal adhesion, cell-substrate junction, and coiled-coil domain (Figure 14A). Similarly, the similar genes were mainly enriched in extracellular matrix structural constituent of molecular function (MF), growth factor binding, and platelet-derived growth factor binding (Figure 14A). In KEGG pathway analysis, similar genes were found to be involved in the Hedgehog signaling pathway, extracellular matrix (ECM)-receptor interaction, and Wnt-activated receptor activity (Figure 14B). GSEA analysis identified mechanisms associated with GLI1 (Figure 14C), GLI2 (Figure 14D), GLI3 (Figure 14E), and GLI1/2/3 gene sets in different cancers, where they were primarily linked to EMT activation.

### Single-cell analysis

**Figure 15:**
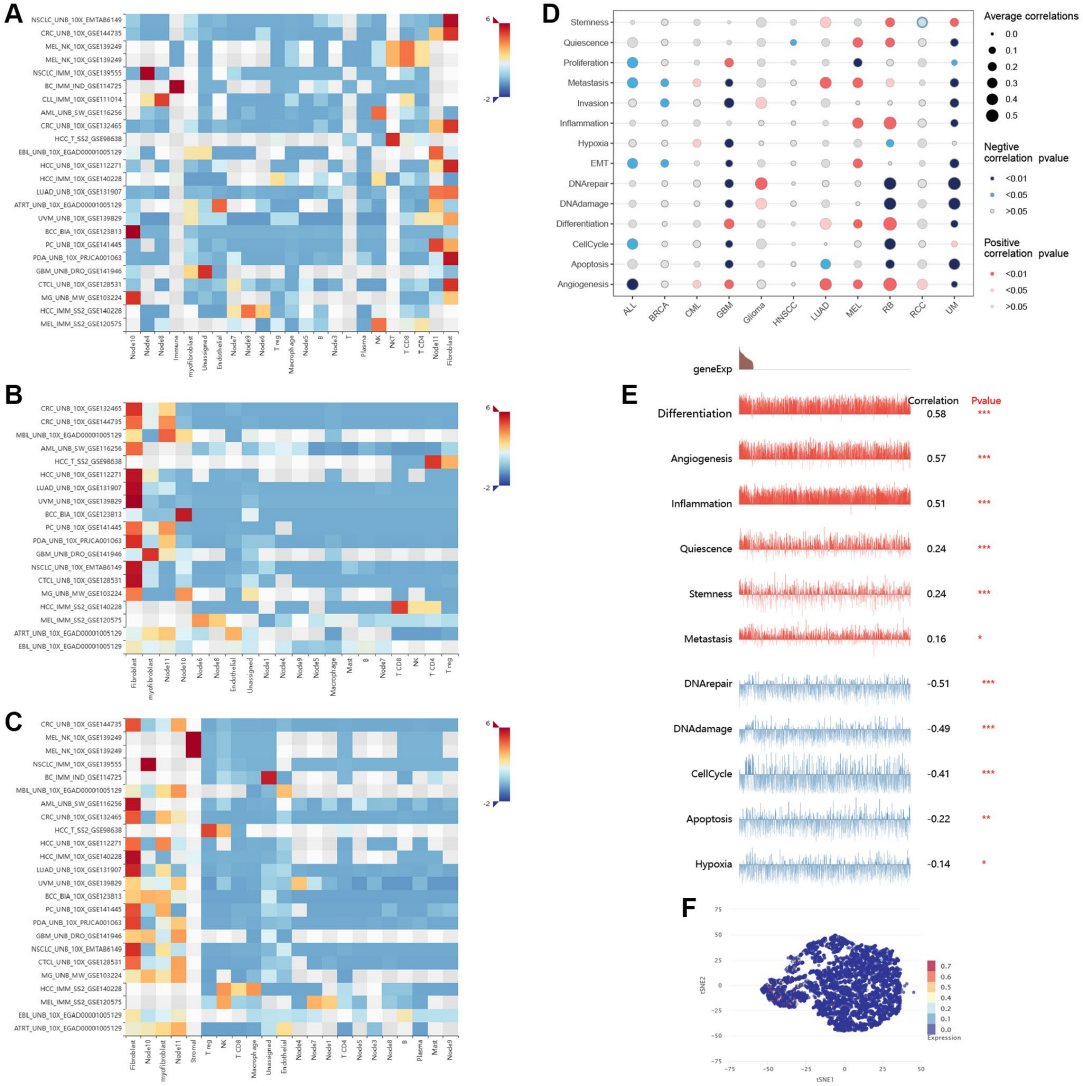
**Single-cell analysis.** The expression distribution of GLI1 (**A**), GLI2 (**B**), and GLI3 (**C**) at the single-cell level. The correlation between the gene set of GLI1/2/3 and cancer-related functional states (**D**). The correlation between the gene set of GLI1/2/3 and cancer-related functional states in RB (**E**). The expression distribution of the gene set of GLI1/2/3 with t-SNE plot in RB is displayed (**F**). T-SNE describes the distribution of cells, where each point represents a single cell, and the color of the point indicates the expression level of the gene list in that cell.

By utilizing different cancer single-cell datasets, we gained an understanding of the expression distribution of GLI1 (Figure 15A), GLI2 (Figure 15B), and GLI3 (Figure 15C) at the single-cell level and found that GLI1, GLI2, and GLI3 were mainly highly expressed in fibroblasts. By integrating single-cell datasets, we analyzed the correlation between the gene set of GLI1/2/3 and cancer-related functional states (Figure 15D), and found that there was a correlation between the gene set of GLI1/2/3 and different cancers and functional states. Figure 15E specifically demonstrates the correlation between the gene set of GLI1/2/3 and cancer-related functional states in RB. Figure 15F displays the expression distribution of the gene set of GLI1/2/3 with t-SNE plot in RB.

## DISCUSSION

Biomarkers are closely associated with disease progression [21–24]. In particular, immune-related biomarkers are most relevant to tumor progression [25, 26]. While much attention has been given to the study of GLI1, the role of GLI2 and GLI3 in tumors has received relatively less attention. Previous research reports have suggested that the aberrant activation of the Hh-GLI signaling pathway may contribute to tumor initiation and progression by enhancing cancer cell proliferation, stimulating stromal activation, and promoting angiogenesis [27]. Furthermore, other signaling pathways such as the mitogen-activated protein kinase (MAPK) cascade, transforming growth factor β (TGF-β) pathway, and epidermal growth factor receptor (EGFR) pathway can also intersect and excessively activate GLI proteins [28]. GLI proteins induce and support tumor development through the regulation of transcription of various oncogenic factors [29]. GLI1 and GLI2 have been found to have overlapping effects on tumor development, while the relationship between GLI3 and the former two is not yet fully understood [30–33]. To investigate the correlation between GLI1/2/3, we conducted correlation line analyses in tumor tissues, normal tissues, and tumor cell lines. Our results showed significant correlations among them in most tumor and normal tissues, but less so in tumor cell lines, possibly due to their higher expression levels in tumor and normal tissues and lower expression levels in cell lines. The significant correlation observed in tumor tissues underscores the importance of studying their roles in cancer. Moreover, our findings indicate that GLI1/2/3 are significantly expressed in most tumors, and their high expression levels are associated with poor prognosis.

GLI1 enhances tumor drug resistance through inducible glucuronidation [34]. Downregulation of GLI1 in colorectal cancer inhibits cancer cell proliferation while involving activation of Wnt signaling [35]. In a study of advanced lung adenocarcinoma, GLI1 mRNA and GLI3 mRNA expression were associated with lower 5-year survival rates in patients [36]. In gastric cancer, GLI2 has been identified as an intrinsic regulator of programmed cell death ligand 1 (PD-L1) expression in tumor cells that promote tumor growth by inhibiting the anti-tumor response [37]. Human epidermal growth factor receptor 2 (HER2) -mediated GLI2 stabilization promotes anoikis resistance and metastasis of breast cancer cells [38]. Enhanced GLI2 transcriptional activity promotes tumor angiogenesis and cancer stemness in renal cell carcinoma [39]. PI3K/AKT can activate GLI1 and GLI2 through the non-classical Hh signaling pathway to enhance renal cell carcinoma's proliferation and clonogenic ability. Hence, the combined inhibition of GLI1 and GLI2 using the GANT61 inhibitor and AKT inhibitor Perifosine has been shown to inhibit cancer cell growth and promote apoptosis [33]. GLI3 inhibition decreases stemness, cell proliferation, and invasion in oral squamous cell carcinoma [40]. Excessive activation of GLI3 promotes prostate cancer growth [41]. Downregulation of GLI3 expression mediates chemotherapy resistance in acute myeloid leukemia [42]. Furthermore, we observed low mutation rates in GLI1/2/3, indicating that their mutations are not major factors in tumorigenesis. We conducted preliminary investigations of their genetic alterations by analyzing the correlations between GLI1/2/3 and CNV, methylation patterns, MMR genes, and methyltransferases. Our findings suggest that inhibiting GLI activity could interfere with almost all types of DNA repair in human cancers, underscoring the potential importance of Hh/GLI function in enabling tumor cells to resist potentially lethal DNA damage induced by chemotherapy and radiation therapy.[43].

We conducted an immune infiltration analysis of GLI1/2/3, and our results revealed their strongest positive correlation with cancer-associated fibroblasts (CAFs) in tumors, followed by endothelial cells and macrophages. Furthermore, correlations with different T cells were also observed. CAFs in primary and metastatic tumors are highly versatile, plastic, and resilient cells that actively contribute to cancer progression through complex interactions with other cell types in the tumor microenvironment. CAFs undergo epigenetic alterations and release secretory factors, exosomes, and metabolites that affect tumor angiogenesis, immunology, and metabolism. These processes are in addition to the production of extracellular matrix components that contribute to the structure and function of the tumor stroma.[44]. It is a key player in the tumor immune microenvironment [5]. CAFs may serve as a potential source of Hh ligands in the tumor stroma of oral squamous cell carcinoma. Moreover, CAFs may respond to Hh signaling by activating nuclear GLI-1, thereby promoting its translocation into the nucleus [45]. Extraterminal domain (BET) proteins have been found to promote the growth of pancreatic ductal adenocarcinoma (PDAC) cells by directly interacting with members of the GLI transcription factor family and regulating their activity. Inhibition of the BET bromodomain reduces the content of tumor-associated fibroblasts in the tumor [46]. By inhibiting Hh signaling, the proliferation of CAF could be inhibited, and tumor immune tolerance and drug resistance could be attenuated [47]. Endothelial cells and fibroblasts in tumors can work together to enhance tumor angiogenesis and act as amplifiers [48]. Macrophages have a critical role in maintaining physiological homeostasis and immunity. However, in the context of cancer, they lose their protective function and become tumor-associated macrophages (TAMs), promoting tumor growth and invasion [49]. This is accompanied by an elevation in immunosuppressive TGF levels in the peritumor skin [50]. In this context, the Hh effector and zinc finger transcription factor GLI2 can directly activate TGFβ expression in human Treg cells [51]. These findings suggest that GLI2 may impair T-cell activation and function by altering the gene expression profile in these cells. Moreover, GLI1/2/3 were found to be positively correlated with immune regulatory genes and checkpoint genes in most tumors. These results suggest that GLI1/2/3 likely plays a role in cancer progression and prognosis through interactions with the tumor microenvironment. Notably, TMB and MSI have been associated with cancer immunotherapy [52]. Analysis of GLI1/2/3 and their correlation further extends their role in immunity.

To explore the impact of the GLI family on cancer, we investigated how the GLI1, GLI2, GLI3, and GLI1/2/3 gene sets affect tumors. Surprisingly, our findings revealed that GLI1, GLI2, and GLI3 promote EMT in most tumors, with the GLI1/2/3 gene set amplifying this effect. EMT remodels cell-cell and cell-extracellular matrix interactions, separating epithelial cells from each other and the underlying basement membrane while activating a novel transcriptional program to promote mesenchymal fate. In the context of tumors, EMT gives cancer cells a greater potential for tumor initiation and metastasis, as well as greater resistance to immunotherapy [53]. Interfering with GLI1/2 expression inhibits TGF-β1-induced EMT in neuroblastoma cells [54]. Pcaf inhibits hepatocellular carcinoma metastasis by targeting GLI-1 to suppress EMT [55]. Hedgehog-GLI (Hh-GLI) promotes EMT in lung squamous cell carcinoma [56]. Suppression of GLI1 expression may inhibit the migration and invasion of HCC cells by down-regulating the expression and activation of MMP-2 and MMP-9, as well as blocking EMT [57]. EMT cells increase breast cancer metastasis through paracrine GLI activation in adjacent tumor cells [58]. The VEGF-C/NRP2/GLI axis is a novel and conserved paracrine means by which EMT cells promote proliferation, migration, and invasion of epithelial breast cancer cells [59]. GLI1 facilitates the EMT induced by TGF-β1 in gastric cancer [60]. Overexpression of SASH1 in hepatocellular carcinoma cells was found to inhibit SHH, SMO, PTC, and GLI1 expression, thereby reducing proliferation, migration/invasion, and EMT progression [61]. The function of Hh-GLI is necessary for the growth, recurrence, and metastasis of human colon cancer xenografts. This pathway induces an epithelial-to-mesenchymal transition, forming solid tumors [62]. Therefore, further studies on the mechanism by which GLI1/2/3 affects tumors via EMT are warranted.

Most medicines currently being tested to inhibit Hh signaling in clinical settings target Smo proteins as their primary focus. However, the development of Smo inhibitors is limited by molecular abnormalities downstream of the Smo level in the Hh signaling pathway and cross-activation of other pathways with the Hh signaling pathway [63]. Since GLI1/2/3 are downstream transcription factors of the Hh signaling pathway, investigating drugs that target them is essential. By combining drug and gene expression data from the CTRP and GDSC databases, we have successfully identified drugs that can jointly target multiple members of this gene set. This approach is distinct from using a single drug to target a single gene. The recent approval of the first Hh inhibitor, glasdegib, for use in combination with low-dose cytarabine highlights the clinically relevant role of the Hh-GLI signaling pathway in treating acute myeloid leukemia (AML) patients unsuitable for high-dose chemotherapy. The therapeutic efficacy of this combination therapy underscores the importance of targeting the Hh-GLI signaling pathway in this fatal leukemia [64]. The combination therapy of HDAC1 and aPKC inhibitors has been shown to prevent GLI1 nuclear maturation and effectively antagonize basal cell carcinoma (BCC) at lower doses than either agent alone. Specifically, an ATP-competitive small molecule inhibitor of aPKC, used in conjunction with vorinostat, is highly effective in disrupting the aPKC-HDAC1 axis in somatic cell carcinoma [65]. PI3K-mTORC1 inhibitor NVP BEZ235 (BEZ235) treatment of OE19 cells resulted in GLI1 and Ihh downregulation. It reduces OE19 cell survival *in vitro*, which can be utilized to treat esophageal cancer [66]. The combination of NVP-LDE-225 and NVP-BEZ-235 was found to inhibit the transcription and expression of GLI in glioblastoma-initiating cells (GICs). Compared to single agents alone, this combination showed superior efficacy in inhibiting tumor growth, regulating the expression of pluripotency-promoting factors and stem cell markers, modulating cell cycle and proliferation, as well as regulating EMT [67].

In summary, our findings in pan-cancer studies suggest that GLI1/2/3 primarily play a pro-cancer role. Immune microenvironment mapping reveals that they are associated with cancer-related fibroblasts, endothelial cells, and macrophages, serving as immunosuppressants. Moreover, they can promote cancer progression through EMT. Targeting GLI1/2/3 may represent a promising therapeutic approach for treating cancer patients in the future.

## MATERIALS AND METHODS

### Correlation analysis between GLI1/2/3

Data on tumor tissue, normal tissue, and tumor cell lines were collected from the UCSC Xena (https://xena.ucsc.edu/), Genotype-Tissue Expression (GTEx; https://gtexportal.org), and Cancer Cell Line Encyclopedia (CCLE; https://sites.broadinstitute.org/ccle/datasets) databases. Tumor tissue data was obtained from The Cancer Genome Atlas (TCGA; https://www.cancer.gov/about-nci/organization/ccg/research/structural-genomics/tcga) database within the UCSC Xena database. Correlation coefficients between GLI1 and GLI2, GLI1 and GLI3, and GLI2 and GLI3 were calculated using the Spearman algorithm for normal tissues, tumor cell lines, and tumor tissues, respectively, with *p*-values < 0.05 considered statistically significant.

### Expression and prognostic analysis of GLI1/2/3

Many studies have been conducted to explore cancer-related biomarkers using the TCGA database [68–71]. We conducted a comprehensive analysis of GLI1/2/3 expression levels in normal tissues, tumor cell lines, and tumor tissues using data from GTEx, CCLE, and TCGA databases, respectively. Additionally, we analyzed the differential expression of GLI1/2/3 between tumor and normal tissues by combining TCGA tumor data and GTEx normal tissue data. Paired tumor tissues and normal tissues from TCGA were used to analyze the differential expression levels of GLI1/2/3 between paired samples. Protein expression profiles of human tissue types were obtained from the Human Protein Atlas (HPA) database (Human Protein Atlas https://proteinatlas.org), and we analyzed the protein expression level of GLI1 using this database. Immunofluorescence staining images were also used to demonstrate the subcellular localization of GLI1, GLI2, and GLI3 in cancer cells. We employed univariate Cox (unicox) analysis to evaluate their predictive performance with respect to OS, DSS, and PFI. UniCox analysis is commonly used to establish the predictive profile of tumor-associated biomarkers in various studies [72–74]. In addition, Kaplan-Meier analysis was used to assess the effect of GLI1 on OS, and the expression of GLI1/2/3 at different stages was further analyzed.

### Mutation profiles of GLI1/2/3

The cBioPortal (https://www.cbioportal.org/) database facilitates the exploration of multidimensional cancer gene set data, allowing visual analysis of different genes, samples, and data types [75]. Detailed mutation information for GLI1/2/3 was analyzed using this database. The impact of mutations on OS was also analyzed.

### CNV, methylation, and mutation frequency analysis

We analyzed the CNV type, CNV expression, methylation level, and mutation frequency of GLI1/2/3 in different tumors based on genetic information from the TCGA database. We also utilized the Spearman algorithm to further analyze the correlation of GLI1/2/3 mRNA expression with CNV and methylation.

### Immuno-infiltration analysis

The TIMER2.0 database (http://timer.cistrome.org/) is utilized to evaluate immune cell infiltration in tumor tissue using RNA-Seq expression profiling data and analyze the correlation between gene expression and immune cells. We have downloaded the correlation between immune cells and GLI1/2/3 in various cancers from the TIMER2.0 database, and visualized the results using the “ggplot2” R package. The Spearman algorithm was employed to calculate the GSVA score for GLI1/2/3 and determine the correlation between the GSVA score and immune cells [76]. Furthermore, the ESTIMATES R package calculates ESTIMATEScore, ImmuneScore, and StromalScore for each tumor. Using the Spearman algorithm, we analyzed the correlation between these three scores and GLI1/2/3.

### Correlation analysis of GLI1/2/3 and other genes

We calculated the correlations between GLI1/2/3 and immune-related genes, and further analyzed their correlations with methyltransferase and MMR genes.

### Correlation analysis of GLI1/2/3 with TMB and MSI

Based on the TCGA data, we calculated the TMB and MSI for each tumor sample. TMB is a measurement of the number of somatic mutations per megabase of the genome, while MSI reflects a defect in DNA mismatch repair mechanisms that result in the accumulation of errors in repetitive sequences. We employed the Spearman algorithm to calculate the correlations between GLI1/2/3 and TMB/MSI. These analyses can provide insights into the potential relationship between HH-GLI signaling pathway activation and genomic instability in various types of cancer.

### Drug sensitivity analysis

We utilized drug and gene expression data from the Cancer Therapeutics Response Portal (CTRP; https://portals.broadinstitute.org/ctrp/) and Genomics of Drug Sensitivity in Cancer (GDSC; https://www.cancerrxgene.org) databases to identify drugs that could co-target GLI1/2/3. Specifically, the top 30 drugs that could co-target GLI1/2/3 were identified using gene set cancer analysis (GSCA; http://bioinfo.life.hust.edu.cn/GSCA) [77]. We analyzed the prognostic value of GLI1, GLI2, GLI3 in the GSE135222 and IMvigor210 datasets using Biomarker Exploration of Solid Tumors database(https://rookieutopia.com/app_direct/BEST/).

### Enrichment analysis

We used the GEPIA2 (gepia2.cancer-pku.cn) database to search for genes associated with GLI1/2/3 in 33 different tumors. Additionally, we performed GO and KEGG analyses using the clusterprofiler R package. To further analyze the gene sets, we calculated enrichment scores separately for GLI1, GLI2, GLI3, and GLI1/2/3 using GSVA and categorized them into high and low expression groups based on median scores. Background gene sets for enrichment analysis were obtained from hallmark gene sets. Differential analysis of samples from the high and low expression groups was then conducted using the limma R package. Finally, GSEA analysis was performed to identify potential biological pathways that may be affected by GLI1/2/3 expression [78].

### Single-cell analysis

To analyze the expression of GLI1/2/3 at the single-cell level, we used the IMMUcan SingleCell RNAseq Database (https://immucanscdb.vital-it.ch/) to analyze their expression in different cancer single-cell datasets. Furthermore, we also utilized the CancerSEA database (http://biocc.hrbmu.edu.cn/CancerSEA/home.jsp) to investigate the correlation between the gene set of GLI1/2/3 and 14 cancer-related functional states. CancerSEA is a multifunctional website aimed at comprehensively exploring different functional states of cancer cells at the single-cell level, covering 14 cellular functional states including angiogenesis, apoptosis, cell cycle, differentiation, DNA damage, DNA repair, EMT, hypoxia, inflammation, invasion, metastasis, proliferation, quiescence, stemness.

## Supplementary Materials

Supplementary Figure 1
