# Supplementary material for: The comprehensive landscape of prognosis, immunity, and function of the GLI family by pan-cancer and single-cell analysis
Source: Aging (Albany NY). 2024 Mar 18;16(6):5123–48. doi: 10.18632/aging.205630 (PMC11006459; doi:10.18632/aging.205630)
Supplement: Supplementary Figure 1 [file aging-16-205630-s001.pdf]

SUPPLEMENTARY FIGURE

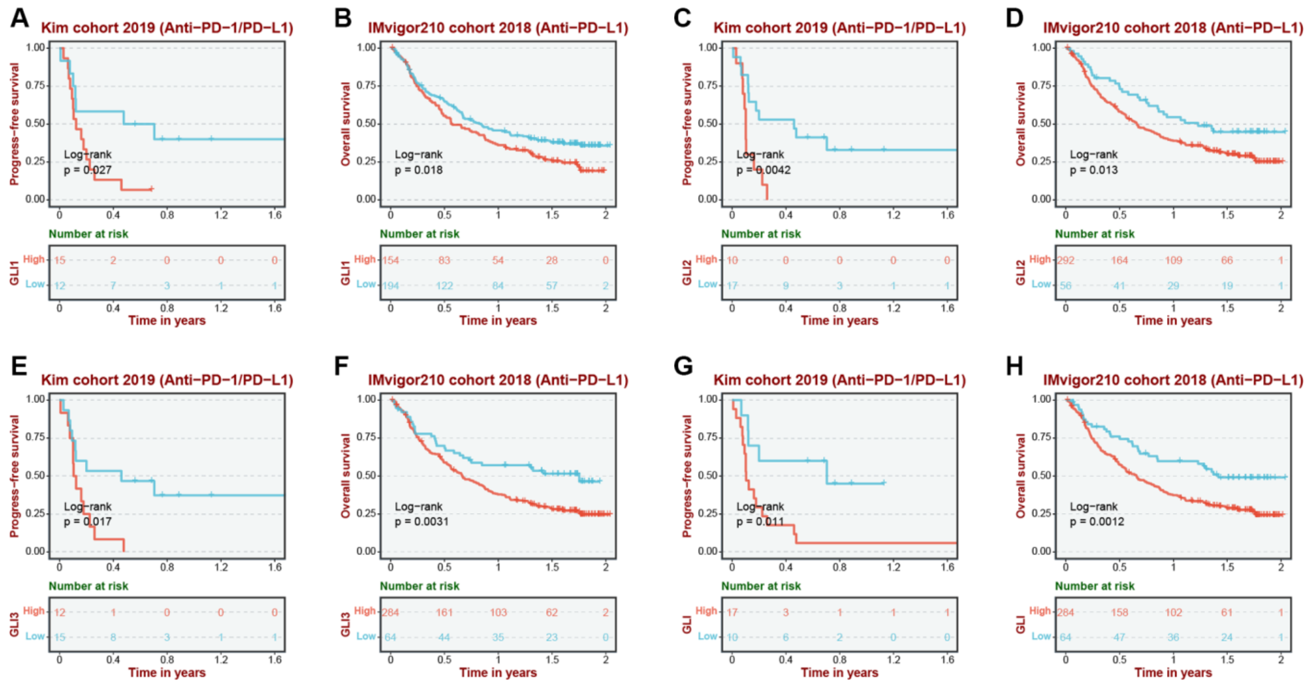

Supplementary Figure 1. Prognostic analysis of immunotherapy cohort. Prognostic analysis of GLI1 (**A**, **B**), GLI2 (**C**, **D**), GLI3 (**E**, **F**), GLI1/2/3 gene set (**G**, **H**) in Kim cohort 2019 (Anti−PD−1/PD−L1) and IMvigor210 cohort 2018 (Anti−PD−L1).
